# Change in Image Quality According to the 3D Locations of a CBCT Phantom

**DOI:** 10.1371/journal.pone.0153884

**Published:** 2016-04-19

**Authors:** Jae Joon Hwang, Hyok Park, Ho-Gul Jeong, Sang-Sun Han

**Affiliations:** Department of Oral and Maxillofacial Radiology, College of Dentistry, Yonsei University, Seoul, Korea; University of North Carolina at Chapel Hill, UNITED STATES

## Abstract

A patient’s position changes in every CBCT scan despite patient alignment protocols. However, there have been studies to determine image quality differences when an object is located at the center of the field of view (FOV). To evaluate changes in the image quality of the CBCT scan according to different object positions, the image quality indexes of the Alphard 3030 (Alphard Roentgen Ind., Ltd., Kyoto, Japan) and the Rayscan Symphony (RAY Ind., Ltd., Suwon, Korea) were measured using the Quart DVT_AP phantom at the center of the FOV and 6 peripheral positions under four types of exposure conditions. Anterior, posterior, right, left, upper, and lower positions 1 cm offset from the center of the FOV were used for the peripheral positions. We evaluated and compared the voxel size, homogeneity, contrast to noise ratio (CNR), and the 10% point of the modulation transfer function (MTF10%) of the center and periphery. Because the voxel size, which is determined by the Nyquist frequency, was within tolerance, other image quality indexes were not influenced by the voxel size. For the CNR, homogeneity, and MTF10%, there were peripheral positions which showed considerable differences with statistical significance. The average difference between the center and periphery was up to 31.27% (CNR), 70.49% (homogeneity), and 13.64% (MTF10%). Homogeneity was under tolerance at some of the peripheral locations. Because the CNR, homogeneity, and MTF10% were significantly affected by positional changes of the phantom, an object’s position can influence the interpretation of follow up CBCT images. Therefore, efforts to locate the object in the same position are important.

## Introduction

CBCT has become the most widely used imaging modality for preoperative diagnosis, and postoperative evaluation of various fields in the dental area. CBCT has the advantage of having a higher resolution, lower dose, and cheaper price than the multi-slice computed tomography (MSCT).[1,2] Additionally, efforts to standardize the CBCT measurement of image quality have been made for the development of a CBCT phantom, including the SEDENTEXCT project[3] from Europe and the DIN 6868–161.[4] Yet, there has been no unified global standard for CBCT image quality assessment until now. Several papers have reported image quality differences according to different CBCT machines, field of views (FOVs), and exposure protocols.[2,5–7] However, these studies only measured the image quality of the object positioned at the center of the FOV. Bamba et al.[8] reported that the noise level was different in the thin slices of two CBCT units when the phantom was located at one peripheral position; however, this study did not discern the effect of the object location 3-dimensionally. There have been no reports analyzing the change in image quality according to the 3D locations using the CBCT phantom until now. The purpose of this study is to evaluate the changes in the image quality according to the 3D phantom locations in the four exposure conditions using the Quart DVT_AP phantom.

## Materials and Methods

### Measurements

**Fig 1:**
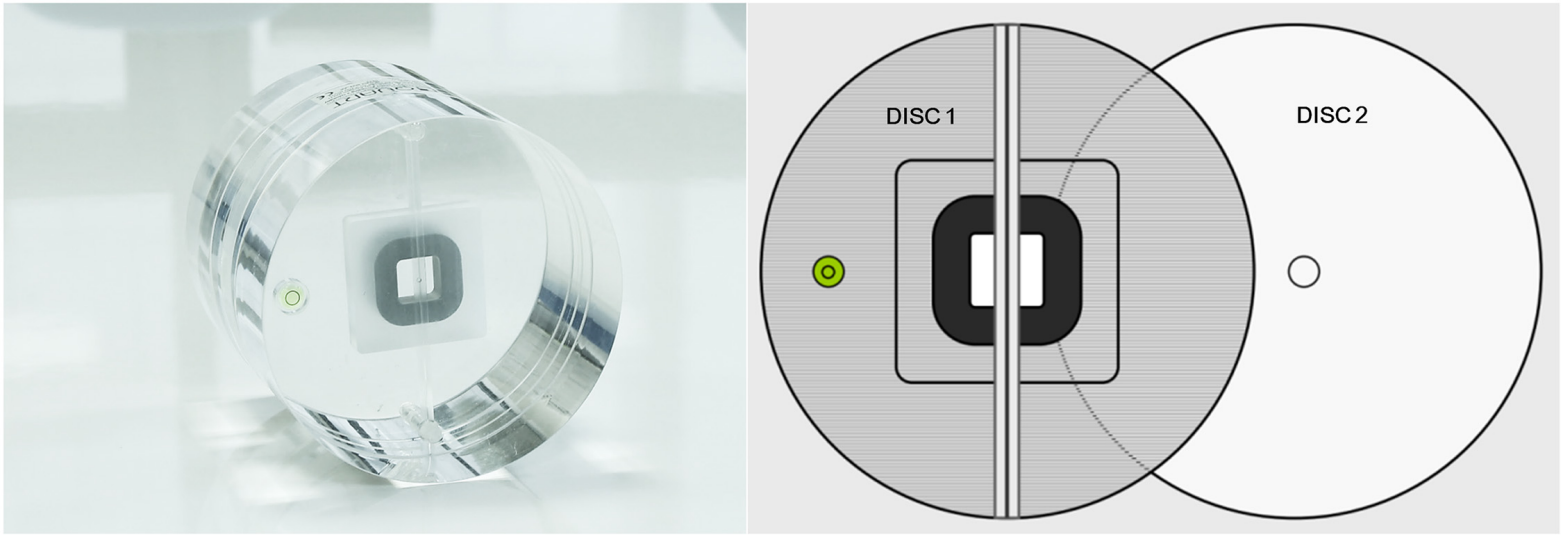
DVT_AP phantom. (A) the DVT_AP phantom. (B) disc 1 containing the test objects and disc 2 containing scatter radiation parts.

**Fig 2:**
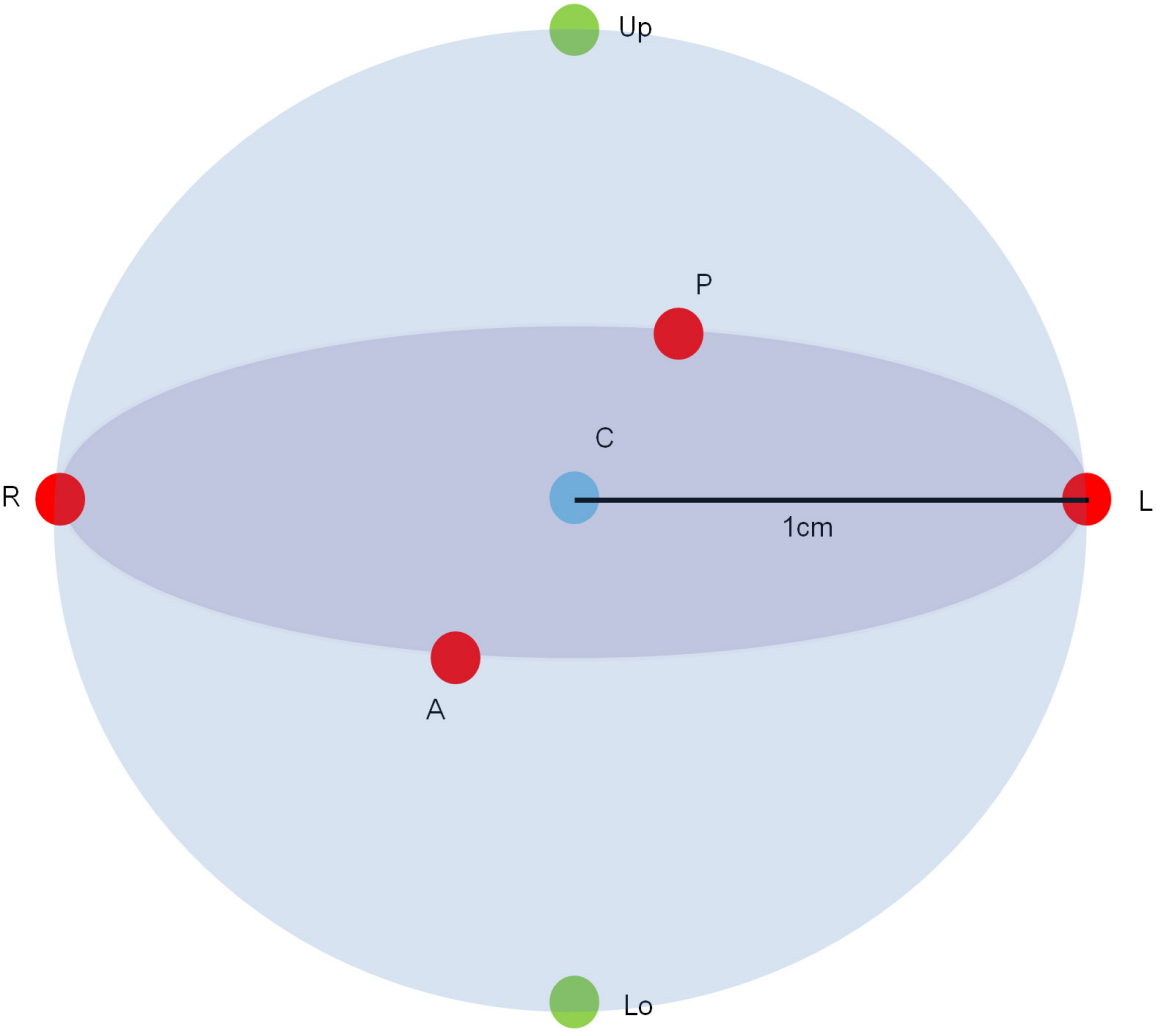
3D locations of the phantom. C represent the center of the FOV; A, P, R, L, Up, and Lo represent anterior, posterior, right, left, upper, and lower positions, respectively, 1 cm offset from the FOV center.

CBCT data were collected using the Rayscan Symphony (RAY Ind., Ltd., Suwon, Korea) and three exposure protocols from the Alphard 3030 (Alphard Roentgen Ind., Ltd., Kyoto, Japan). The exposure protocols used for each CBCT unit are shown in Table 1. The QUART DVT_AP (QUART GmbH, Zorneding, Germany) is made of polymethyl methacrylate (PMMA) containing all of the required test objects for quality control (Fig 1). The center of the FOV was used as a reference position according to the phantom manual. The center of the Quart DVT_AP phantom was located in the upper, lower, right, left, anterior, and posterior positions 1 cm offset from the center of the FOV (Fig 2). All of the examinations were repeated five times independently. Because there is no ear holder and head rest in the Symphony, these components were removed from the Alphard in order to maintain similar examination conditions. One of the protocols with the smallest FOV of the Alphard was excluded because the images of the test object were partially obtained in the peripheral positions and some indexes could not be obtained.

**Table 1:** Cone beam CT machines and exposure protocols.

CBCT Device	Mode	FOV [diameter(mm) x height(mm)]	Scan mode	Voltage (kV)	Current (mA)	Exposure time(s)	Voxel size (mm)
**Alphard 3030**	**Implant (I)**	102 x 102	Full	80	8mA	17s	0.20
	**Panorama (P)**	154 x 154	Full	80	5mA	17s	0.30
	**Cephalo (C)**	200 x 200	Full	80	5mA	17s	0.39
**Ray Symphony**		142 x 142	Full	90	10mA	13s	0.38

**Fig 3:**
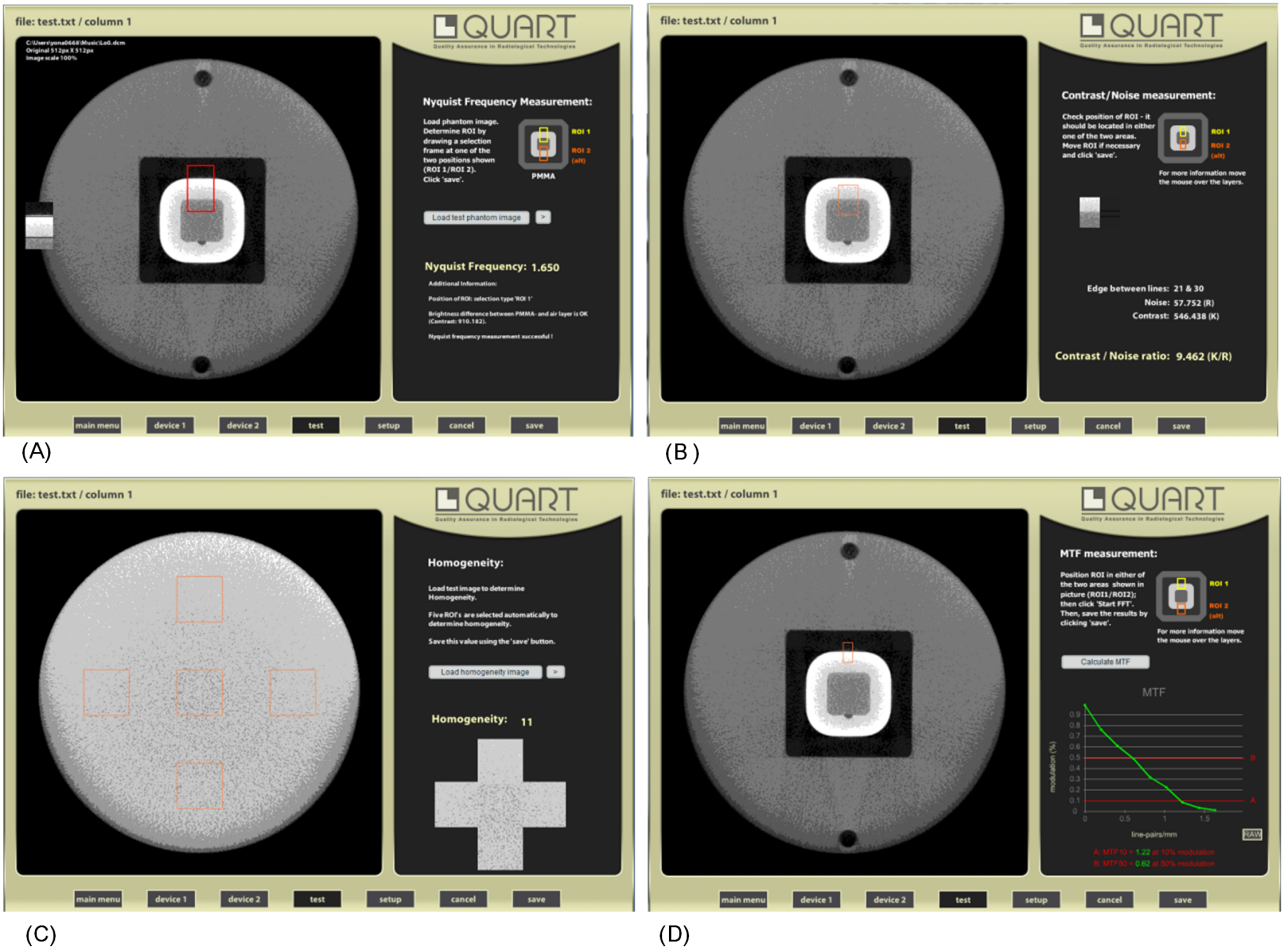
QUART DVT_TEC software for image quality assessment. (A) Nyquist Frequency. (NF) (B) Contrast to Noise Ratio (CNR). (C) Homogeneity. (D) Modulation Transfer Function (MTF). “Screenshots from QUART DVT_TEC software under a CC BY license, with permission from QUART GmbH, original copyright 2014.”

All CBCT images were stored using the DICOM 3.0 (512 X 512 pixel) into a Windows 7-based workstation (Intel Core i5 3570, 4 GByte, calibrated 21.3 inch color monitor, resolution 1563 X 2048 pixels, NVIDIA Quadro 2000 graphics card) and transferred to the QUART DVT_TEC (QUART GmbH, Zorneding, Germany) software for semi-automatic image quality evaluation (Fig 3). Image quality assessments were measured twice by one experienced radiologist for intra-observer reliability.

### Image quality assessment items

Four image quality indexes were analyzed using the Quart DVT_AP phantom and QUART DVT_TEC software as follows. All tolerance levels are from the phantom manual (Table 2):

**Table 2:** Definition and tolerance of four image quality indexes. All tolerance levels are from the phantom manual.

Image quality indexes	Definition	Tolerance
**Voxel size (Nominal resolution)**	unit pixel dimension (mm)	5% of the manufacturer's specification
**Contrast to noise ratio (CNR)**	a ratio of contrast between the PVC-PMMA and the mean image noise of the PVC-PMMA	20% of the manufacturer's specification
**Homogeneity**	relationship between the measured basic contrast and measured background change within a homogenous slice	> 5.0
**10% point of the modulation transfer function (MTF10%)**	the spatial frequency where the modulation is 10%	≥ 1.0

The voxel size (nominal resolution) is the unit pixel dimension (mm) obtained from the Nyquist frequency (NF). The NF is defined as the nominal resolution capability of an x-ray system. The NF is a value independent from dose intensity and object location. The voxel size within 5% of the manufacturer's specifications is defined as the recommended tolerance.

The CNR is defined as a ratio of contrast between the PVC-PMMA and the mean image noise of the PVC-PMMA. It describes to what extent a visual contrast can be discerned from the statistical background variations. A CNR within 20% of the manufacturer's specifications is defined as the recommended tolerance range. Because there were no CNR specifications provided by both manufacturers, the acceptability of the CNR could not be evaluated directly in this study. Instead, the CNR of the center and periphery was compared.

Homogeneity is defined as the relationship between the measured basic contrast and measured background change within a homogenous slice. The homogeneity is measured in a slice image of disc2. A homogeneity value over 5.0 is defined as the recommended tolerance.

The MTF (modulation transfer function) is defined as a measure of how the x-ray system reproduces details of an object in the image. Spatial resolution is usually represented by an MTF10%, which is the spatial frequency where the modulation is 10%. The value is given in line pairs per millimeter (Lp/mm). An MTF10% above 1.0 is defined as the tolerance level.

### Statistical analysis

Because the manual selection of the ROI was preceded in the process of image quality assessment by use of the QUART DVT_TEC software, the intra-class correlation coefficient (ICC) was calculated for evaluating the reliability of manual processing. Image quality indexes of six peripheral locations were statistically compared with those of the center position using the Wilcoxon signed-rank test (p<0.05). Statistical analysis was carried out with the SPSS 20.0 (SPSS Inc., Chicago). A tolerance of all indexes was assessed, too.

## Results

**Fig 4:**
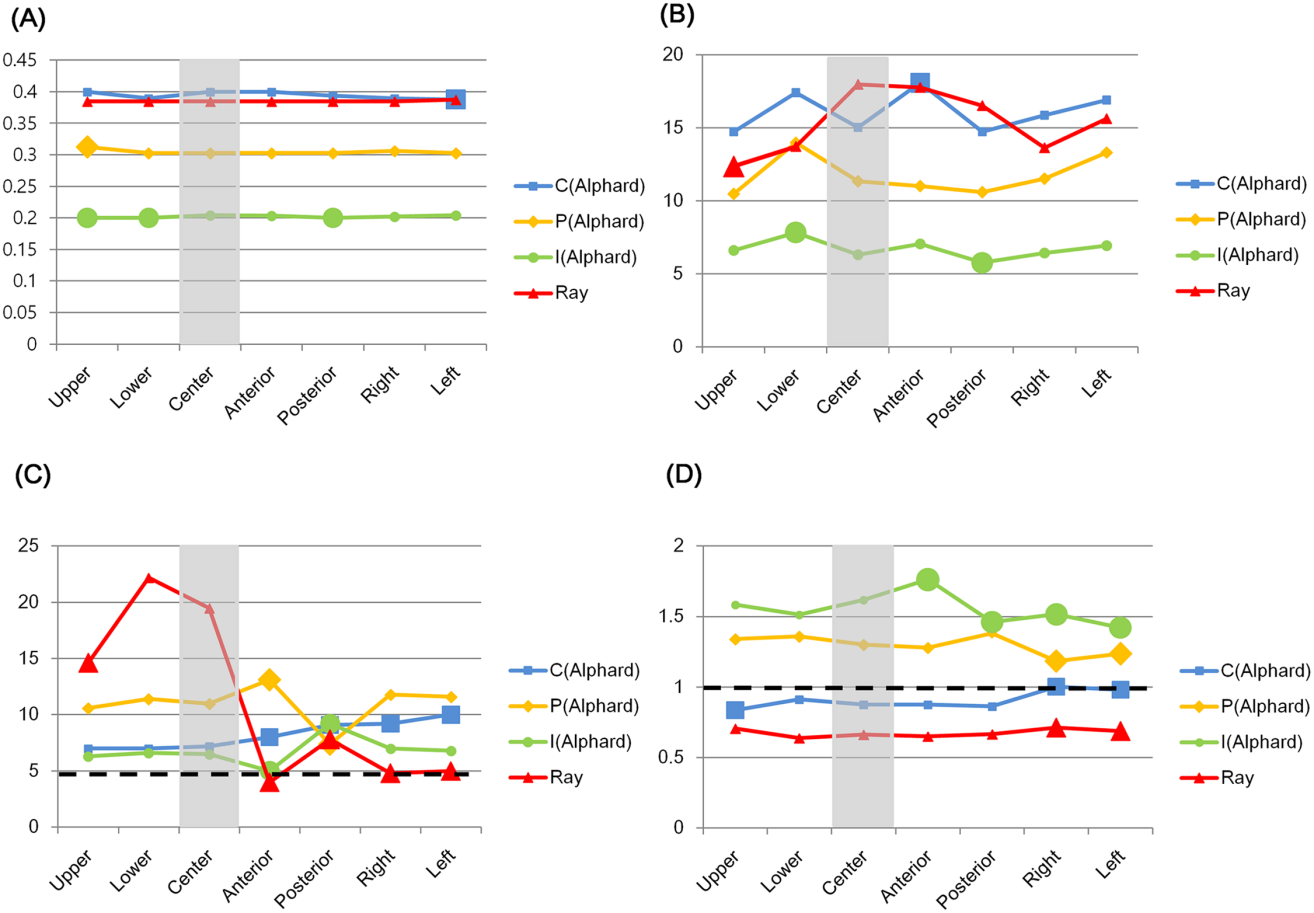
Statistical analysis of image quality index values. (A) Voxel size. (B) Contrast to Noise Ratio (CNR). (C) Homogeneity. (D) 10% point of the modulation transfer function (MTF10%). C, P, and I represent cephalo, panorama, and implant modes of the Alphard 3030 CBCT, respectively. Ray is the Ray Symphony CBCT. Gray columns represent the center position, large nodes represent the values with statistically significant difference compared with the center, and black dotted lines represent the tolerance levels.

The ICC of the image quality indexes were all above 0.96. Image quality indexes among the five repeated images taken from the same location showed different means and standard deviations. These values were varied according to the exposure protocols and the phantom’s location (Table 3). Four image quality indexes showed statistically significant differences at some of the peripheral locations. Moreover, those locations were different for each image quality index (Table 4, Fig 4).

**Table 3:** Means and standard deviations (SD) of four indexes. SD is the standard deviation. CNR = Contrast to Noise Ratio. MTF10% = the 10% point of the modulation transfer function. Upper, Lower, Center, Anterior, Posterior, Right, and Left represent upper, lower, center, anterior, posterior, right, and left positions 1 cm offset from the center of the FOV. Measurements below the tolerance are shown in bold. The CNR, which showed over 20% difference with the center, is also marked in bold.

Machine	Mode	Index	Mean ± SD	Mean ± SD						
				Upper	Lower	Center	Anterior	Posterior	Right	Left
**Alphard 3030**	Implant (I)	Voxel size	0.20 ±0.00	0.20 ±0.00	0.20 ±0.00	0.20 ±0.00	0.20 ±0.00	0.20 ±0.00	0.20 ±0.00	0.20 ±0.00
		CNR	6.71 ±0.65	6.60 ±0.50	**7.83** ±0.87	6.31 ±0.53	7.06 ±0.73	5.76 ±0.48	6.44 ±0.58	6.94 ±0.96
		Homogeneity	6.81 ±1.28	6.40 ±0.55	6.60 ±0.55	6.60 ±0.55	**5.00** ±0.00	9.30 ±0.45	7.00 ±0.00	6.80 ±0.45
		MTF10%	1.56 ±0.11	1.60 ±0.04	1.51 ±0.06	1.60 ±0.06	1.77 ±0.16	1.47 ±0.07	1.52 ±0.03	1.43 ±0.07
	Panorama (P)	Voxel size	0.30 ±0.00	0.31 ±0.00	0.30 ±0.00	0.30 ±0.00	0.30 ±0.00	0.30 ±0.00	0.31 ±0.00	0.30 ±0.00
		CNR	11.74 ±1.79	10.46 ±0.98	**13.99** ±1.17	11.34 ±1.64	11.00 ±0.49	10.58 ±1.59	11.50 ±1.97	13.30 ±1.24
		Homogeneity	10.96 ±1.72	10.60 ±0.55	11.40 ±0.55	11.00 ±0.00	13.10 ±0.22	7.40 ±0.55	11.80 ±0.84	11.40 ±0.55
		MTF10%	1.30 ±0.08	1.34 ±0.06	1.36 ±0.02	1.30 ±0.06	1.28 ±0.04	1.38 ±0.05	1.18 ±0.02	1.23 ±0.02
	Cephalo (C)	Voxel size	0.39 ±0.01	0.40 ±0.00	0.39 ±0.01	0.40 ±0.00	0.40 ±0.00	0.39 ±0.01	0.39 ±0.01	0.39 ±0.01
		CNR	16.11 ±2.00	14.72 ±1.88	17.42 ±2.03	15.02 ±1.29	**18.08** ±2.00	14.74 ±1.06	15.87 ±1.83	16.90 ±1.44
		Homogeneity	8.21 ±1.17	7.00 ±0.00	7.00 ±0.00	7.20 ±0.45	8.00 ±0.00	9.10 ±0.22	9.20 ±0.45	10.00 ±0.00
		MTF10%	0.91 ±0.07	**0.84** ±0.01	**0.91** ±0.04	**0.88** ±0.01	**0.88** ±0.05	**0.86** ±0.02	1.00 ±0.03	**0.99** ±0.04
**Ray Symphony**		Voxel size	0.39 ±0.00	0.38 ±0.00	0.38 ±0.00	0.38 ±0.00	0.38 ±0.00	0.38 ±0.00	0.38 ±0.00	0.39 ±0.01
		CNR	15.37 ±3.12	**12.35** ±2.05	**13.71** ±4.01	17.97 ±1.80	17.77 ±2.43	16.52 ±3.09	**13.64** ±2.02	15.62 ±1.87
		Homogeneity	11.13 ±7.17	14.60 ±0.55	22.20 ±0.84	19.50 ±2.24	**4.00** ±0.00	7.80 ±0.45	**4.80** ±0.45	**5.00** ±0.00
		MTF10%	0.67 ±0.03	**0.71** ±0.04	**0.64** ±0.02	**0.66** ±0.02	**0.65** ±0.01	**0.67** ±0.02	**0.71** ±0.02	**0.69** ±0.02

**Table 4:** Statistical analysis of image quality index values. ^a^ represents p<0.05. CNR = Contrast to Noise Ratio. MTF10% = the 10% point of the modulation transfer function. Upper, Lower, Center, Anterior, Posterior, Right, and Left represent upper, lower, center, anterior, posterior, right, and left positions 1 cm offset from the center of the FOV. Measurements below the tolerance are shown in bold. The CNR, which showed over 20% difference with the center, is also marked in bold.

Machine	Mode	Index	P value					
			Upper	Lower	Anterior	Posterior	Right	Left
**Alphard 3030**	Implant (I)	Voxel size	.025^a^	.025^a^	.157	.025^a^	.157	1.000
		CNR	.500	**.043**^a^	.138	.043^a^	.893	.345
		Homogeneity	.564	1.000	**.038**^a^	.039^a^	.157	.564
		MTF10%	.893	.138	.043^a^	.043^a^	.043^a^	.043^a^
	Panorama (P)	Voxel size	.025^a^	1.000	1.000	1.000	.180	1.000
		CNR	.345	**.080**	.317	.345	.686	.080
		Homogeneity	.157	.157	.034^a^	.038^a^	.102	.180
		MTF10%	.225	.138	.686	.080	.043^a^	.043^a^
	Cephalo (C)	Voxel size	1.000	.059	1.000	.157	.059	.046^a^
		CNR	.686	.080	**.043**^a^	.686	.500	.138
		Homogeneity	.317	.317	.046^a^	.039^a^	.025^a^	.034^a^
		MTF10%	**.043**^**a**^	**.080**	**.893**	**.068**	.043^a^	**.043**^a^
**Ray Symphony**		Voxel size	1.000	1.000	1.000	1.000	1.000	.317
		CNR	**.043**^**a**^	.138	.500	.500	**.080**	.080
		Homogeneity	.043^a^	.080	**.043**^a^	.043^a^	**.043**^**a**^	**.043**^a^
		MTF10%	**.136**	**.138**	**.686**	**.686**	**.043**^**a**^	**.043**^a^

### Voxel size (nominal resolution)

Voxel sizes of all of the exposure protocols were within tolerance, which was 5% of the manufacturer’s specification (Table 3). The voxel size of the Alphard showed statistically significant differences at some of the peripheral locations. The voxel size of the Symphony did not show any statistically significant difference (Table 4, Fig 4(A)).

### CNR

The CNR of both CBCTs showed statistically significant differences at some of the peripheral locations. These locations were the lower and posterior positions of the Alphard implant (I) mode, the anterior position of the Alphard cephalo (C) mode and the upper and posterior positions of the Symphony (Table 4, Fig 4(B)). Among these, there were positions which showed mean differences over 20% with the center, which was 24.09% in the lower position of the Alphard I mode, 20.37% in the anterior position of the Alphard C mode and -31.27% in the upper position of the Symphony (Tables 4 and 5).

**Table 5:** The mean difference (%) with the center position. CNR = Contrast to Noise Ratio. MTF10% = the 10% point of the modulation transfer function. Upper, Lower, Center, Anterior, Posterior, Right, and Left represent upper, lower, center, anterior, posterior, right, and left positions 1 cm offset from the center of the FOV. Measurements below the tolerance are shown in bold. The CNR, which showed over 20% difference with the center, is also marked in bold.

Machine	Mode	Index	Upper (%)	Lower (%)	Anterior (%)	Posterior (%)	Right (%)	Left (%)
**Alphard 3030**	Implant (I)	Voxel size	0.00	0.00	0.00	0.00	0.00	0.00
		CNR	4.60	**24.09**	11.89	-8.72	2.06	9.98
		Homogeneity	-3.03	0.00	**-24.24**	40.91	6.06	3.03
		MTF10%	0.00	-5.63	10.63	-8.13	-5.00	-10.63
	Panorama (P)	Voxel size	3.33	0.00	0.00	0.00	3.33	0.00
		CNR	-7.76	**23.37**	-3.00	-6.70	1.41	17.28
		Homogeneity	-3.64	3.64	19.09	-32.73	7.27	3.64
		MTF10%	3.08	4.62	-1.54	6.15	-9.23	-5.38
	Cephalo (C)	Voxel size	0.00	-2.50	0.00	-2.50	-2.50	-2.50
		CNR	-2.00	15.98	**20.37**	-1.86	5.66	12.52
		Homogeneity	-2.78	-2.78	**11.11**	**26.39**	**27.78**	**38.89**
		MTF10%	**-4.55**	**3.41**	**0.00**	**-2.27**	13.64	**12.50**
**Ray Symphony**		Voxel size	0.00	0.00	0.00	0.00	0.00	2.63
		CNR	**-31.27**	**-23.71**	-1.11	-8.07	**-24.10**	-13.08
		Homogeneity	-25.13	13.85	**-79.49**	-60.00	**-75.38**	**-74.36**
		MTF10%	**7.58**	**-3.03**	**-1.52**	**1.52**	**7.58**	**4.55**

### Homogeneity

Homogeneity of the center was within tolerance in all exposure protocols (Table 3, Fig 4(C)). Meanwhile, homogeneity of both CBCTs showed statistically significant differences at some peripheral locations. Especially, the anterior and posterior locations of all exposure protocols showed statistically significant differences compared to the center. There were other locations that showed statistically significant differences, which were the right and left positions of the Alphard C mode and the upper, right, and left positions of the Symphony (Table 4, Fig 4(C)). Among these, the anterior of the Alphard I mode and the anterior, right, and left positions of the Symphony showed values under tolerance (~5%) (Table 3, Fig 4(C)). The biggest mean difference with the center was 40.91% of the Alphard I mode and -79.49% of the Symphony (Table 5).

### MTF10%

The Alphard C mode and Symphony showed the MTF10% under the tolerance in all positions except the right position of the Alphard C mode. Other modes of the Alphard showed the MTF10% within tolerance (Table 3, Fig 4(D)). The MTF10% of both CBCTs showed statistically significant differences at some of the peripheral locations. Especially, the right and left positions of both CBCTs showed statistically significant differences. There were other locations which showed statistically significant differences, which were the anterior and posterior positions of the Alphard I mode and the upper position of the Alphard C mode (Table 4, Fig 4(D)). The biggest mean difference with the center was 13.64% of the Alphard I mode and 7.58% of the Symphony (Table 5).

## Discussion

Previous studies reported a gray value (GV) variation according to the location inside the object.[9–11] These studies assumed an ideal condition that the object was positioned at the center of the FOV; yet this is different from clinical situations where patients are not always at the same position. Other studies showed a GV change according to the phantom location.[12–15] However, analyzing a GV difference is one quantitative approach from the perspective of each voxel, and it is difficult to obtain the overall information about the change in image quality, which can be represented by various indexes including the CNR, homogeneity, MTF10% and so on.

The three main physical factors affecting image quality are now generally considered to be the contrast, noise, and sharpness [16], which are represented by the CNR, homogeneity, and MTF, respectively. Each factor is related to unique features of the anatomical structures expressed by various imaging modalities. The CNR is related to the imaging performance with respect to large, low-contrast structures [17,18], whereas the MTF is related to the reproduction of small structures. [17,19] Homogeneity is related to the noise distribution and affects the low-contrast detectability. [18,20]

The three indexes above also describe the physical performance of the imaging system in specific conditions, but it is often difficult to link these to clinical performance.[16] Nonetheless, there have been reports about the correlation between the MTF [21], CNR [21–28], and subjective evaluation in lesion detection. The CNR can be an important factor in the evaluation of the periodontal ligament space and lamina dura [21], which are important for the periapical and malignant lesion detection in dental diagnostic radiology.[29–31] Additionally, there was a report that a higher CNR made a higher correlation between the CBCT and micro-CT in bone quality evaluation for implant installation.[32] Homogeneity is also reported to affect the lesion detection performance.[28] The MTF is reported to affect the sensitivity for detection of root fractures.[33–35] On the basis of these correlation with subjective and clinical evaluations, the phantom study has been used as a pre-requisite for obtaining quality images in the clinical setting, currently.[36]

In spite of increased attention about a correlation between objective and subjective image quality evaluation, there has been no study yet about the image quality needed for comparing follow up CBCT images. Comparing current images with previous ones is important for lesion detection. However, even experienced radiologists sometimes fail to interpret the images correctly.[37,38] To reduce this failure, physical factors that can affect the comparison performance need to be analyzed beforehand. Following the same line of thought, analyzing the effect of the change in object position should precede the comparison of follow up CBCT images.

In this study, the voxel size showed statistically significant differences at some of the peripheral locations. Because the voxel sizes of all of the exposure protocols were within the tolerance (~5%), these differences were not substantially important. This is because the signals obtained from the CBCT detector are sampled by an NF fixed for the specific exposure protocol. This result showed that the voxel sizes were all acceptable in this experiment, and other image quality indexes were nearly influenced by the voxel size.

The CNR, homogeneity, and MTF10% of both CBCTs showed statistically significant differences at some of the peripheral locations (Table 4, Fig 4). The average differences between the center and these peripheries were up to 31.27% (CNR), 70.49% (homogeneity), and 13.64% (MTF10%) (Table 5). The different tolerance levels for image quality are reportedly needed according to the treatment such as bone quality evaluation for implants and periapical lesion detection.[21] Therefore, in order to compare follow up CBCT images accurately, different tolerance levels of image quality might also be required. Furthermore, considerable image quality differences according to the object’s location might affect the complex and time-consuming comparison procedure. Image quality difference also can influence image subtraction [39,40], which has significantly raised the accuracy of the lesion detection.[41–43]

The European Commission in EUR 16262 defined image quality criteria as a level of performance considered necessary to produce images of standard quality for a particular anatomical structure.[44] Therefore, image quality indexes below the tolerance might affect the interpretation of an anatomical structure. In this study, homogeneity was below tolerance at some of the peripheral locations. There have been studies which have reported that there was little correlation between homogeneity and lesion detection.[21,45] However, homogeneity in those studies was all within the tolerance and there was no lesion size classification. Another study reported that the lesion detection performance was influenced by homogeneity when the lesion size was below 1 mm.[46] When comparing follow up CBCT images, radiologists often make efforts to identify the subtle changes of the lesion size or bone pattern around the lesion. Therefore, unacceptable homogeneities might affect the lesion detection performance of follow up CBCT images.

The C mode of the Alphard 3030 and Symphony showed an MTF10% below the tolerance except at one peripheral position (the left position of the Alphard C mode) (Table 3, Fig 4D). Many of the MTF10% of the CBCT with a large voxel size (about 0.4 mm) were below tolerance in some studies.[1,47,48] However, an MTF10% above 1.0 is recommended as a realistic spatial resolution of the CBCT [1] and this is possible in an exposure protocol with a voxel size of about 0.4 mm, as this result showed. Therefore, an acceptable MTF10% of the center has to be guaranteed first before the proper evaluation of the MTF10% at the peripheral positions.

The patient position has been an important issue in diagnostic radiology because the form of the internal organs can be influenced by different patient postures.[49–51] However, the effect of the position itself has not been thoroughly reviewed in previous studies. In this study, different object positions resulted in significant and considerable changes in the image quality indexes that might affect the comparison of follow up CBCT images. This result is meaningful because any CBCT image may be compared for other purposes such as lesion detection or post-implantation checkups. Therefore, it is important for radiologists and technicians to seek a more reproducible patient positioning protocol.

This study has the limitation of a physical experiment that analyzed image quality indexes using a CBCT phantom. Therefore, an additional study using a jawbone or cadaver should be needed for evaluating the effect of the position change on the subjective evaluation of the follow up CBCT images. Due to use of the QUART DVT_TEC software, it was impossible to analyze the rotated and tilted images. In a future study, it is necessary to evaluate rotated and tilted images for reproducing clinical situations.

## Conclusions

Because the CNR, homogeneity, and MTF10% were significantly affected by positional changes of the phantom, an object’s position can influence the interpretation of follow up CBCT images. Therefore, efforts to locate the object in the same position are important.

## Supporting Information

S1 Document Granted permission (the screenshots of the DVTtec). (PDF)

S1 Table Experiment data (C mode of the Alphard 3030). (XLSX)

S2 Table Experiment data (P mode of the Alphard 3030). (XLSX)

S3 Table Experiment data (I mode of the Alphard 3030). (XLSX)

S4 Table Experiment data (Ray Symphony). (XLSX)
