# Supplementary material for: Change in Image Quality According to the 3D Locations of a CBCT Phantom
Source: PLoS One. 2016 Apr 19;11(4):e0153884. doi: 10.1371/journal.pone.0153884 (PMC4836729; doi:10.1371/journal.pone.0153884)
Supplement: S1 Document — (PDF) [file pone.0153884.s001.pdf]

QUART GmbH ▪ Kirchenweg 7 ▪ D-85604 Zorneding

QUART
Quality Assurance in Radiological Technologies

To: James Hwang.
Department of Oral and Maxillofacial Radiology,
College of Dentistry,
Yonsei University,
Seoul, Korea

QUART *Medizintechnische Geräte* GmbH
- Kirchenweg 7
  D-85604 Zorneding
- Tel.: +49 (0) 8106 / 249 118
- Fax: +49 (0) 8106 / 249 119
- www.quart.biz
- info@quart.biz

Zorneding, 15th February 2016

Subject: Permission to reproduce images of DVTtec under a CCAL

Dear James Hwang,

I acknowledge receipt of your mail related to the above subject.

I can confirm that we grant you the permission to use the images included (in low resolution) in the appendix to this letter under the creative commons attribution license (CCAL) CC BY 4.0.

The images could be included in your PLOS ONE publication on condition that it includes the following acknowledgment where appropriate:

"The author thanks the company QUART GmbH for permission to reproduce the screenshots of the first version of the software DVTTec. More information on this software can be found in www.quart.de. QUART has no responsibility for the placement and context in which the images are reproduced by the author, nor is QUART in any way responsible for the other content or accuracy therein".

As with such permission, we would require if possible copy of the published paper paper for our files.

Please contact me if you have any further questions.

Best wishes,

Hugo de las Heras Gala
Research and communication
QUART GmbH
85604 Zorneding, Germany

hugo@quart.de

Appendix

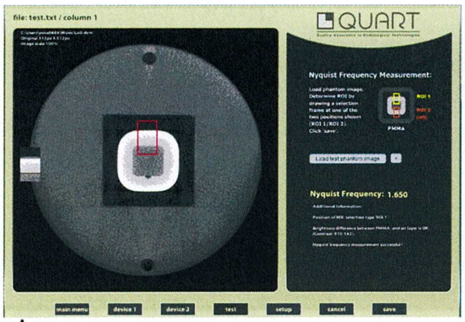

A

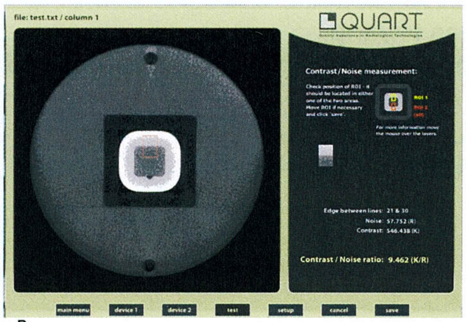

B

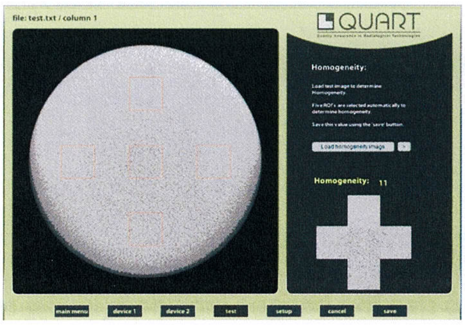

C

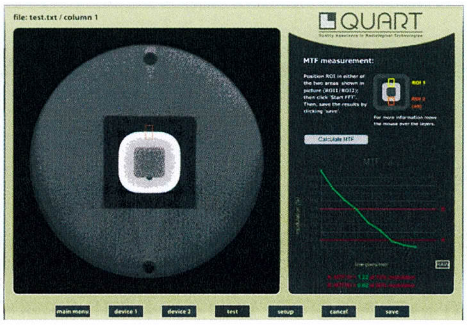

D
